# Characterization and Pathogenicity of the Novel Porcine Reproductive and Respiratory Syndrome Virus 1 Strain SL-01 in China

**DOI:** 10.1155/2024/6873468

**Published:** 2024-05-15

**Authors:** Jiaying Zheng, Yu Wu, Xiaopeng Gao, Limiao Lin, Hao Chang, Guojun Zhu, Siyun Fang, Wei Li, Bohua Ren, Qunhui Li, Xiangbin Zhang

**Affiliations:** ^1^College of Animal Science, South China Agricultural University and Guangdong Provincial Key Lab of Agro-Animal Genomics and Molecular Breeding, Guangzhou 510642, China; ^2^State Key Laboratory of Biocontrol, Guangzhou Higher Education Mega Center, School of Life Sciences, Sun Yat-sen University, Guangzhou 510000, China; ^3^Wen's Food Group, Yunfu 527400, China

## Abstract

Currently, PRRSV-1 causes a large number of clinical infections in Chinese swine herds, and the prevalence of new strains has presented great challenges. In this study, the novel PRRSV-1 strain SL-01 was isolated, with a genome length of 14,978 bp, and genetic evolution analysis revealed that it belonged to a new subtype branch. Sequence homology analysis showed that the strain was only 82.2%–86.7% identical to the current classical PRRSV-1 strains. In particular, the novel strain exhibited a unique deletion pattern in Nsp2. In addition, GP3 and GP4 of the SL-01 strain showed four consecutive amino acid deletions in the highly variable regions at amino acids 243–248 and 63–68, respectively. Further challenges in piglet and pregnant sow demonstrated that the SL-01 strain could cause the piglet fever and death but less pathogenic to pregnant sow. Overall, the characterization and pathogenicity of a novel PRRSV-1 strain were first explored and provide a prevention for pig farms.

## 1. Introduction

Porcine reproductive and respiratory syndrome is a highly infectious disease caused by porcine reproductive and respiratory syndrome virus (PRRSV). This infection mainly leads to piglet death, respiratory symptoms, sow abortion, stillbirth, mummification, and other breeding disorders [1–3]. PRRSV is a positive, single-stranded RNA virus with a genome size of approximately 15 kb [4]. At least 11 open reading frames (ORFs) have been identified, with ORF1a and ORF1b accounting for 80% of the viral genome and encoding the multimeric protein precursors ppla and pplab, respectively [5]. ORF2–ORF7 account for only 20% of the viral genome and encode eight viral structural proteins: GP2a, E, GP3, GP4, GP5, GP5a, M, and N [2, 6].

PRRSV can be divided into types 1 (European genotype) and 2 (North American genotype) [4, 7, 8]. The nucleotide identity between PRRSV-1 and PRRSV-2 is only 55%–70% [9]. The PRRSV-1 Lelystad virus strain was first reported and isolated in the Netherlands in 1991 [10–12]. Subsequently, similar strains were detected in European and American countries as well as Asian countries such as China, Korea, and Thailand [13–15]. In 1997, the PRRSV-1 strain B13 was first detected in China. In 2011, the BJEU06-1 and NMEU09-1 strains were first isolated and reported in Chinese pig farms [16]. To date, PRRSV-1 is endemic in at least 20 Chinese provinces [17]. In recent years, the PRRSV-1 detection rate has gradually increased in China. PRRSV-1 strains reported in China can be divided into four subgroups: NMEU09-1-like, Amervac-like, HKEU16-like, and BJEU06-1-like strains [18, 19].

Studies have shown that PRRSV-1 is generally less pathogenic than PRRSV-2 and causes milder respiratory diseases [20, 21]. In 2017, Italian researchers isolated PRRSV-1 (PR40/2014) with high pathogenicity, which the infected pigs showed severe clinical symptoms such as high fever, respiratory distress, and high mortality [22]. The emergence of PRRSV-1 strains in China is mainly through the pig trade. In 2016, the GZ11-G1 strain, isolated from the pig farm, can cause fever, showing a certain pathogenicity to piglets [23]. The NPUST2789 strain isolated in Taiwan is weakly pathogenic but can induce viremia and damage the lungs in nursery pigs [24]. In 2023, the 181187-2 strain was successfully isolated from serum samples collected, which belongs to the same subtype as the highly pathogenic strain PR40/2014 isolated from Italy and has moderate pathogenicity [25]. In addition, the ZD-1 strain reported in 2023 showed moderate pathogenicity to piglets in clinical samples [26].

Recently, the detection rate of PRRSV-1 in clinical samples has increased significantly, thereby seriously limiting pig breeding performance. Therefore, we monitored and investigated the epidemic variation in PRRSV-1 in China through the isolation and whole-genome sequencing of novel PRRSV-1 strains from clinical samples as well as pathogenic models of piglets and sows.

## 2. Materials and Methods

### 2.1. Sampling and Virus Isolation

The clinical samples were collected from Wen's pig farm, which has 5,000 sows. All pigs had a complete immunization process with VR2332 MLV vaccine in the farm. However, the 21-day-old piglets suddenly died with clinical symptoms being fever, dyspnea, and cyanosis of the skin. The main samples we collected were mainly lung tissue, serum, and whole blood, etc.

Antibiotic-added DMEM was added to lung tissue samples for grinding, and the ground tissue was stored at −80°C for three freeze–thaw cycles. The supernatant was then isolated by centrifuge for viral nucleic acid extraction. For serum and whole blood samples, the supernatant was directly aspirated for RNA extraction. Total RNA was extracted from the supernatant using the RNeasy kit (Magen, China). PRRSV was detected by reverse transcription polymerase chain reaction (RT-PCR). Positive samples were diluted with the medium, seeded with primary alveolar macrophages (PAMs) through a 0.22-*μ*m filter for virus isolation.

### 2.2. RT-PCR Amplification and PRRSV Genome Sequencing

The RNeasy kit was used to extract total RNA from cell culture samples. All primers used to amplify PRRSV genome fragments are designed and stored in our laboratory. RT-PCR was performed using the Prime Script™ RT-PCR kit (Promega, Madison, WI, USA) and PrimeSTAR® GXL DNA polymerase, respectively. The PCR products were cloned into pMD19-T vectors with the TOPO® TA Cloning® kit (Invitrogen, USA) and sent to Sangon Biotech (Shanghai, China) for sequencing. For each amplicon, more than three independent clones are sequenced to accurately sequence a specific genomic region.

### 2.3. Immunofluorescence Assay

PRRSV- or mock-infected cells were washed thrice with PBS, fixed with 4% paraformaldehyde for 15 min at room temperature, and then infiltrated with 0.2% Triton X-100 (Solarbio, Beijing, China) for 15 min. Then, the cells were washed with PBS for thrice, and blocked with 3% bovine serum albumin (Solarbio) for 1 hr at room temperature. Next, cells were incubated overnight with anti-PRRSV-N antibody at 4°C. After three washes with PBS, cells were incubated with anti-mouse IgG secondary antibodies (Cell Signaling Techniques, Danvers, MA, USA) for 45 min at RT. Images were captured and processed using an inverted fluorescence microscope.

### 2.4. Sequencing Analysis

All sequencing results were spliced and aligned using Lasergene SeqMan (DNASTAR, Madison, WI, USA) and MegAlign (DNASTAR). All reference sequences obtained from GenBank were used for sequence alignment and phylogenetic analysis. All phylogenetic trees were constructed using MEGA7.0 software (https://www.megasoftware.net/).

### 2.5. Histopathology

At necropsy, lung tissues were fixed in 10% buffered neutral formalin for hematoxylin and eosin and immunohistochemical staining, as previously described. Dyeing was automatically performed using a Leica fully automatic dyeing machine (Leica, Wetzlar, Germany). The sample slides were observed under a 200x microscope.

### 2.6. Animal Experiment Design

To assess the pathogenicity of the novel PRRSV strain SL-01, we conducted a challenge study in both piglet and pregnant sow. For piglets, 10 healthy piglets aged 35 days were divided into 2 groups (5 piglets per group). The challenge test was performed via an intramuscular injection of 2 ml of PRRSV-1 SL-01 culture medium (10^5^ TCID_50_), whereas the negative control was inoculated with DMEM. Daily rectal temperatures and clinical signs were recorded for each group. Serum samples were collected weekly, and the blood viral load and antibody levels were analyzed. The survival rate of the piglets was recorded during the experiment. All animals were euthanized after the experiment.

For the sow pathogenicity experiment, six pregnant sows were randomly divided into two groups and challenged at 85 days of gestation. After the challenge, the body temperature, viral load, and PRRSV-N antibodies of pregnant sows were detected. Finally, the delivery of pregnant sows was observed and recorded.

### 2.7. Statistical Analysis

In each experimental group, statistical significance was measured using one-way analysis of variance. Two-sided *P*-values of <0.05 were considered to indicate statistical significance.

## 3. Results

### 3.1. Virus Isolation

**Figure 1:**
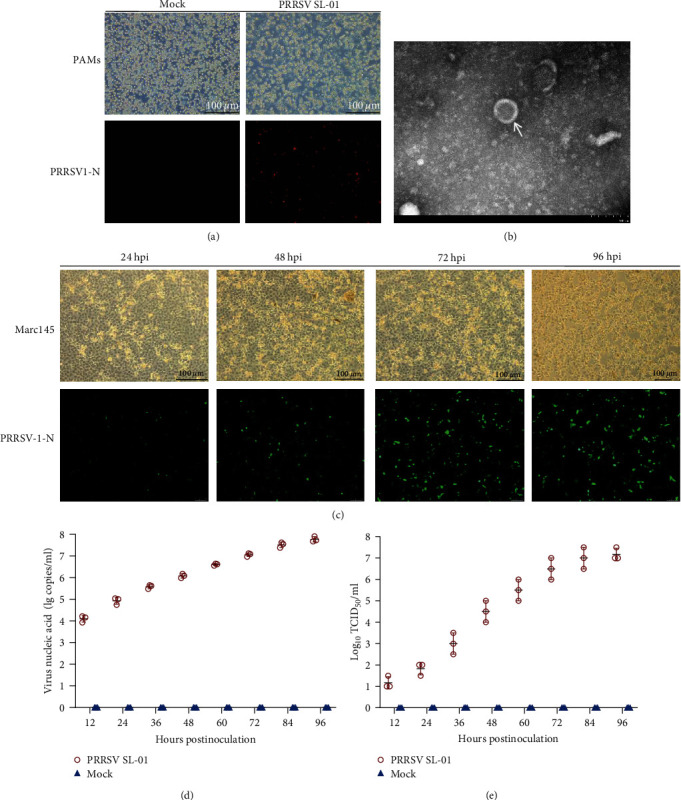
Strain isolation and identification. (a) Virus inoculation in PAMs detected via IFA. (b) Electron micrograph of SL-01 (indicated by arrows). Magnification, 200,000x. (c) Cytopathic conditions and IFA revealing the reactivity of a monoclonal antibody against the PRRSV-N protein from SL-01 at 24, 48, 72, and 96 hr postinfection (hpi). Magnification, 400x. (d) SL-01 viral titer detection at 12, 24, 36, 48, 60, 72, 84, and 96 hpi. (e) Growth kinetics of SL-01.

Positive samples were screened from recently collected clinical samples, and the virus was isolated from PAMs. PAMs showed a stable cytopathic effect, and the PRRSV-N protein was detected via the indirect immunofluorescence assay (IFA) (Figure 1(a)). Further observation of the virion morphology via transmission electron microscopy revealed a virion size of approximately 55 nm and a globular structure (Figure 1(b)), named SL-01. Then, the virus was transferred into Marc-145 cells to assess its biological characteristics. With increasing infection time, cytopathies such as cell death and shedding became more pronounced (Figure 1(c)). Meanwhile, the IFA results revealed an increase in the positive fluorescence signal with culture time (Figure 1(c)). In addition, the detection of virus titer and nucleic acid proliferation revealed that both have a similar increase trend (Figures 1(d) and 1(e)).

### 3.2. Genomic Characterization and Phylogenetic Analysis

The whole gene of the SL-01 strain is about 14,978 bp. Then, seven classical representative PRRSV-1 strains were downloaded from NCBI, and the nucleotide sequence identity of the SL-01 strain was analyzed. Alignment of the full-length genome sequence demonstrated that SL-01 shared 82.2%–86.7% nucleotide identity with the seven representative strains (Table 1).

**Figure 2:**
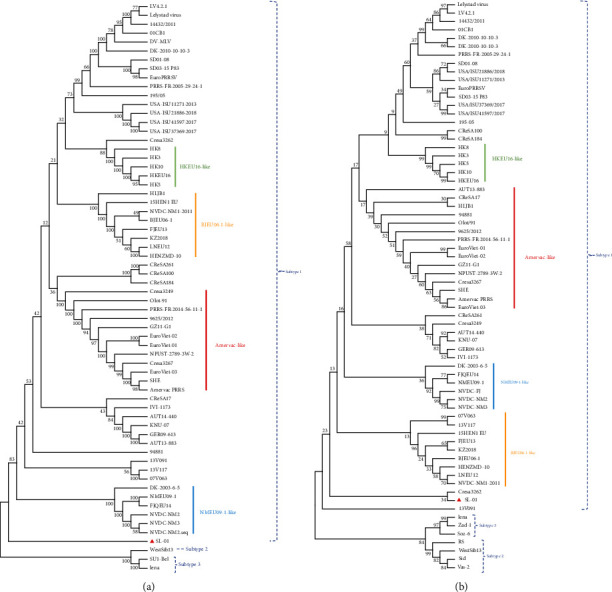
Phylogenetic analysis of SL-01. (a) Phylogenetic trees constructed based on whole-length SL-01 genomes. (b) Phylogenetic trees constructed based on the ORF5 gene of SL-01.

Phylogenetic analysis illustrated that the whole-genome sequence of SL-01 did not belong to any branch of the current subtype, and it was separately classified into a new branch with low homology with the existing subtype strains (Figure 2(a)). Genetic evolutionary analysis based on ORF5 revealed that both SL-01 strains could be classified into separate subtype clades (Figure 2(b)).

### 3.3. Nsp2 Amino Acid Analysis

**Figure 3:**
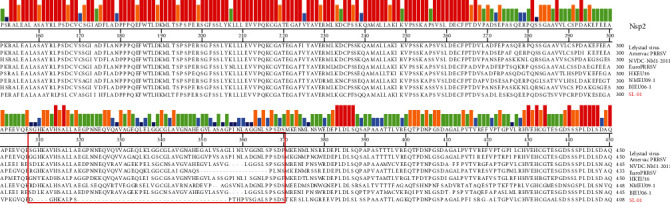
Amino acid sequence alignment based on Nsp2. The results showed four consecutive amino acid deletions in 309–312 and 37 continuous amino acid deletions in 320–355.

The results of Nsp2 amino acid sequence analysis, compared with seven strains classical reference, showed that there were four consecutive amino acid deletions in 309–312 and 37 continuous amino acid deletions in 320–355, indicating that this strain had a large degree of deletion and mutation in Nsp2, which was quite different from the classical strain (Figure 3).

### 3.4. GP3/4 Amino Acid Analysis

**Figure 4:**
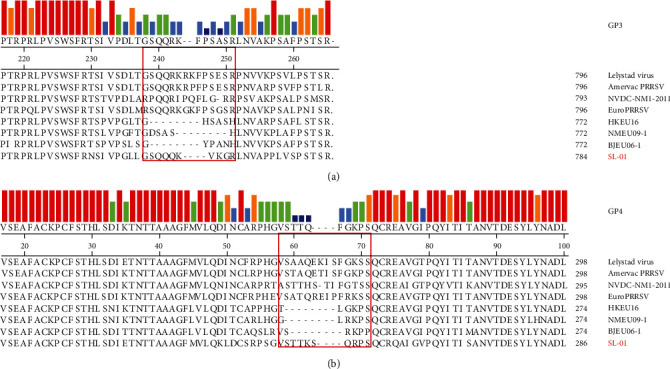
Amino acid sequence alignment based on GP3 and GP4. (a) GP3 had four consecutive amino acid deletions in the highly variable region of 243–248. (b) GP4 also had four consecutive amino acid deletions in 63–68.

Among the structural proteins of PRRSV-1, GP3 and GP4 contain hypervariable regions similar to those of Nsp2, including amino acids 237–252 of GP3 and amino acids 57–72 of GP4. Therefore, we performed amino acid sequence analysis on GP3 and GP4 of the SL-01 strain. The results showed that GP3 had four consecutive amino acid deletions in the highly variable region of amino acids 243–248 (Figure 4(a)), and GP4 also had four consecutive amino acid deletions in 63–68 (Figure 4(b)). Studies have shown that NMEU09-1, HKEU16-l, and BJEU06-1 strains also have similar amino acid deletions [27].

### 3.5. Piglet Pathogenicity Analysis

**Figure 5:**
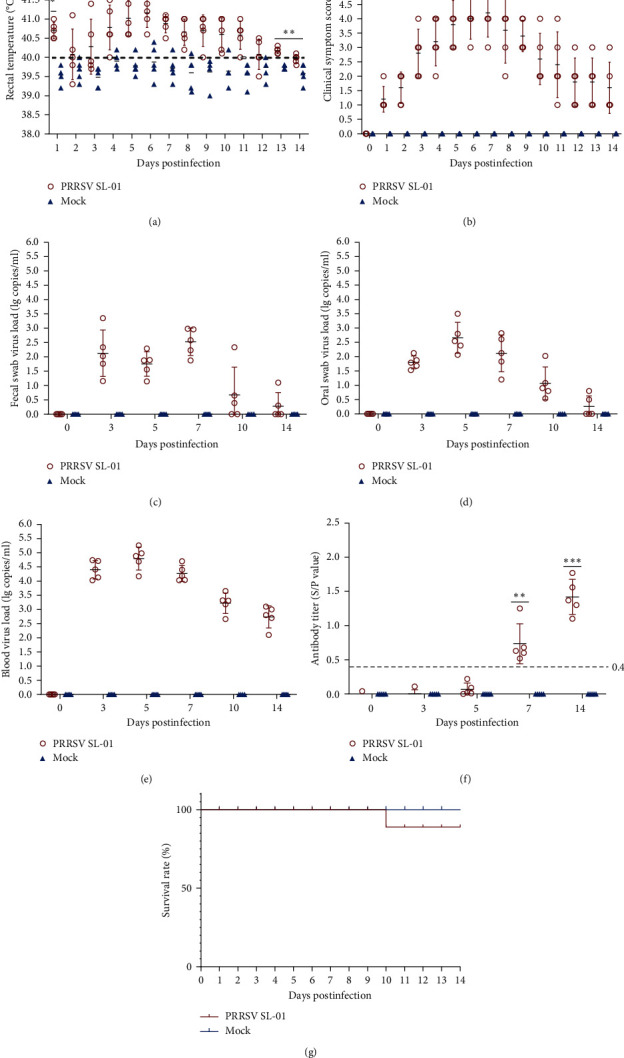
Pathogenicity results in piglets. (a) Body temperature changes in piglets in each group after challenge. (b) Clinical scores of piglets after challenge throughout the experiment. (c–e) Viral load detection in the blood, oral swabs, and pharyngeal swabs. (f) PRRSV-specific antibody level in each group during the challenge study. (g) Survival rate of pigs in each group during the challenge study. Each bar represents the mean ± standard deviation in each group. Significant differences are marked with asterisks:  ^*∗∗∗*^*P* < 0.001,  ^*∗∗*^*P* < 0.01, and  ^*∗*^*P* < 0.05.

The pathogenicity results of piglets showed that the body temperature gradually increased at 3 dpi, reached the highest temperature at 6 dpi, and then gradually returned to normal (Figure 5(a)). During the entire experiment, the body temperature of the SL-01 strain was higher than that of the control group, resulting in fever in pigs. In terms of clinical symptom score, the SL-01 strain was able to cause obvious symptoms in pigs compared with the control group, with the highest score at 7 dpi (Figure 5(b)). In addition, not only was a large viral load detected in the blood, but viral nucleic acid was detected in both oral and fecal swabs, indicating that the SL-01 strain can not only cause high levels of viremia but also detoxify the mouth and feces (Figure 5(c)–5(e)). Further detection of PRRSV-N antibodies in serum found that the SL-01 strain caused positive PRRSV antibodies and produced high levels of antibodies at 14 dpi (Figure 5(f)).

**Figure 6:**
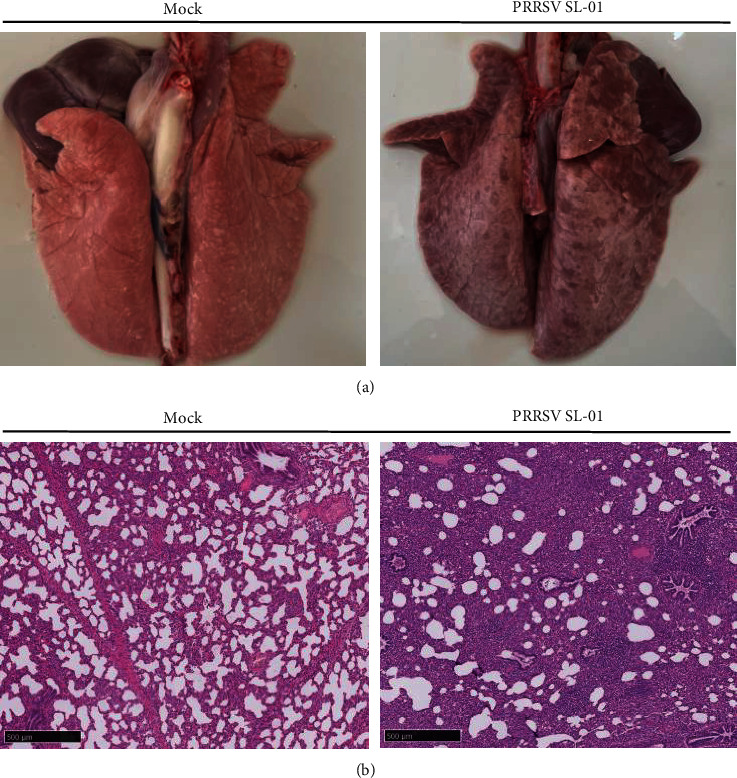
Observation and detection via pathological necropsy. (a) Lung necropsy observation: the lung tissue showed significant consolidation and diffuse hemorrhage after PRRSV SL-01 challenged. (b) Histopathology tests: alveolar cell inflammatory infiltrate.

During the experiment, one pig died at 10 dpi in the SL-01 group and no pig death occurred in the control group. Finally, piglet survival results showed that the piglet survival rate was 80% in the SL-01 group and 100% in the blank control group (Figure 5(g)). At 14 dpi, each group of pigs is euthanized. At autopsy, the SL-01 strain was found to cause localized bleeding in the lungs (Figure 6(a)). Further histopathological examination showed damage to the alveoli after infection, resulting in inflammatory infiltration and thickening of the lungs, while no damage was done in the control group (Figure 6(b)).

### 3.6. Pregnant Sow Pathogenicity Analysis

**Figure 7:**
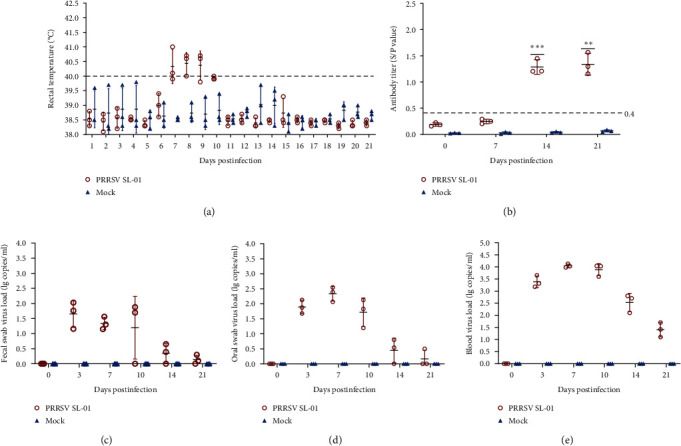
Pathogenicity results in pregnant sows. (a) Body temperature changes in pregnant sows in each group after challenge. (b) PRRSV-specific antibody level in each group during the challenge study. (c–e) Viral load detection in the blood, oral swabs, and pharyngeal swabs. Each bar represents the mean ± standard deviation in each group. Significant differences are marked with asterisks:  ^*∗∗∗*^*P* < 0.001,  ^*∗∗*^*P* < 0.01, and  ^*∗*^*P* < 0.05.

The pathogenicity of the PRRSV-1 SL-01 strain was further evaluated by the pregnant sows. The whole experiment lasted 21 days, and after the challenge, the pregnant sows did not immediately develop fever, only a slight increase in body temperature at 7, 8, 9, and 10 dpi, about 40°C–40.5°C (Figure 7(a)), and then returned to normal. At the same time, pregnant sows did not show other obvious clinical symptoms after challenge. But viral load can still be detected from mouth swabs, swabs, and in the blood (Figure 7(c)–7(e)). Besides, starting from 14 dpi, the PRRSV-N antibody in the SL-01 group gradually increased, while the control group was negative (Figure 7(b)). Finally, the results of delivery data showed that there were no sow deaths and abortions after challenge, but the number of weak litters and stillbirths in the challenge group was higher than that in the control group (Table 2). Meanwhile, the viral nucleic acid of PRRSV-1 was detected in the umbilical cord blood of piglets in the challenge group (Table 2).

## 4. Discussion

In 1997, Chinese Customs intercepted pigs infected with PRRSV-1 [26]. In 2011, the GZ11-G1 strain was isolated, which shares high nucleotide sequence homology with the PRRSV-1 Amervac strain [16, 27]. This could be associated with the introduction of vaccinated breeding pigs [28]. Since then, PRRSV-1 infections have been continuously reported in China [29, 30]. In recent years, the positivity rate of PRRSV-1 strains has increased rapidly. In 2016, 50 samples were collected from 750 breeding farms in Guangdong Province, with the PRRSV-1 positivity rate of 24.8% (186/750) [31, 32]. Moreover, with the recent outbreak of African swine fever virus in China, the surveillance of animal diseases has increased. This increased monitoring has indirectly led to an increase in the number of PRRSV-1 detection reports in China [26]. In this study, the PRRSV-1 strain SL-01 was isolated from pig farms in Guizhou Province. Genetic evolution analysis revealed that SL-01 does not belong to any current subtype, indicating that it represents a new branch and that new subtype strains are beginning to appear and circulate in China.

Nsp2 is the most varied protein among PRRSV nonstructural proteins. PRRSV-1 and PRRSV-2 strains have complex and diverse patterns of amino acid insertion, mutation, and deletion during evolution [33]. In 2011, the mutations in Nsp2 and GP3 were first reported in PRRSV-1 in China [16]. The deletion of these regions is considered a possible biomarker for the PRRSV-1 variant. Moreover, a systematic analysis of Nsp2 indel patterns in 19 Chinese PRRSV-1 strains showed that these strains formed 12 indel patterns. Among them, the deletion of aa 357–360+aa 411 (9/19 viruses) was most common in the Chinese PRRSV 1 strain. In this study, the Nsp2 amino acid analysis of the SL-01 strain showed that there were four consecutive amino acid deletions in 309–312 and 37 continuous amino acid deletions in 320–355. The results showed that the SL-01 strain had a large degree of deletion and mutation in Nsp2, which may be due to differences in the virulence of the strain.

Another hypervariable region other than Nsp2, the ORF3/4 overlap region, is commonly deleted in PRRSV-1 isolates [34]. GP3 is a small strut-310 protein encoded by ORF3 that contains a hypervariable region located at the carboxy terminus of car-311 (amino acids 237–252) of the PRRSV-1 ORF3/4 overlap region [15, 35]. Previous studies illustrated that this region can mutate earlier than other regions under immune selection pressure. Mutation hotspots at approximately amino acids 237–252 and 57–72 of the ORF3/4 overlap region were confirmed [36, 37]. The deletion of eight consecutive amino acids (241–248) represented the most common deletion [19, 34]. In this study, we found that SL-01 had four consecutive amino acid deletions in both GP3 and GP4, similar to previous studies.

Although the number of reported PRRSV-1 strains isolated in China has gradually increased, the pathogenic characteristics of Chinese PRRSV-1 strains remain limited. Previous studies have shown that the GZ11-G1 strain is pathogenic but not fatal to piglets, and it can cause respiratory symptoms and transient increases in body temperature [23]. The HLJB1 strain causes low-level viremia in infected pigs and induces a favorable immune response [37]. In 2023, a latest report revealed that the 181187-2 strain belonged to the same subtype as the highly pathogenic strain PR40/2014 isolated in Italy [25]. The ZD-1 strain is moderately pathogenic in piglets [22, 25]. In this study, assessments of piglets and pregnant sows infected with the SL-01 strain indicated that this strain can cause high body temperature, obvious clinical symptoms, and high-level viremia. Most obviously, SL-01 caused death, resulting in a survival rate of 80%. The pathogenicity of the novel PRRSV-1 strain indicates that SL-01 is more pathogenic than previous epidemic strains. The pathogenic results from pregnant sows demonstrated that SL-01 only causes mild fever in pregnant sows, but it can increase the proportion of weak sows, which affects the productive performance of pregnant sows. Overall, SL-01 was more pathogenic to piglets than to pregnant sows. We speculate that the cause of this pathogenic difference is caused by differences in its genetic sequence and mutations in amino acids, and the specific effects still need further study.

## 5. Conclusion

In this paper, a new subtype of PRRSV-1 strain SL-01 was isolated from clinical samples, which was quite different from the current strain in genetic sequence and had different mutation patterns of key amino acids, which may lead to changes in the virulence of the strain. Further pathogenic models of piglets and sows showed that the strain could cause fever and death in piglets but was less pathogenic to sows. The specific causes of this difference in pathogenicity require further study. In general, a PRRSV-1 strain with high pathogenicity was found in this paper, and its genetic differences were further explained, which provided a reference for clinical monitoring and biosecurity control of the PRRSV-1 strain.

## Figures and Tables

**Table 1:** Nucleotide sequence similarity between SL-01 strain and reference strains.

SL-01	Amervac PRRSV	BJEU06-1	EuroPRRSV	HKEU16	Lelystad virus	NMEU09-1	NVDC-NM1-2011
Complete genome	85.4	84.1	85.4	84.1	86.7	82.2	83.6
ORF1a	84	82.3	84.3	80	86	79.1	81.9
ORF1b	86	84.8	86.6	85.2	87	85.2	84.3
ORF2a	87.7	88	86.9	86.5	88.3	84	85.7
ORF2b	93	94.8	92.5	88.7	93.4	90.1	91.5
ORF3	85.6	84.8	84.7	85	86.6	83.9	83.7
ORF4	83.3	84.1	83	83.7	84.6	84.7	82.5
ORF5	84.7	82	82.8	84.7	84.7	82	83.8
ORF6	91.4	88.9	90.4	90.4	90.4	91.2	88.3
ORF7	87.9	89.4	87.9	89.4	89.4	87.9	88.6

**Table 2:** Sows delivery data after challenge.

Groups	Mock	SL-01
Challenge virus	DMEM	Virus cultures
Piglets, number per litter		
Total born	11/13/15	11/13/10
Live born	9/11/14	8/10/9
Still born	8/10/11	6/5/7
Mummified	0/0/0	1/1/1
Light (<1 kg)	2/2/1	2/3/2
Sow abortion	0/3 (0%)	0/3 (0%)
Sow survival	3/3 (100%)	3/3 (100%)
Fetal PRRSV virus load	Negative	Positive

## Data Availability

The experimental data used to support the findings of this study are available from the corresponding author upon request.
